# Study on Abnormal Angiogenesis in Moyamoya Disease via Mitochondrial D‐Loop Methylation

**DOI:** 10.1002/brb3.71042

**Published:** 2025-11-21

**Authors:** Yuting Luo, Heng Ye, Chutong Guo, Shaoqing Wu, Xunsha Sun

**Affiliations:** ^1^ Department of Neurology The First Affiliated Hospital, Sun Yat‐sen University; Guangdong Provincial Key Laboratory of Diagnosis and Treatment of Major Neurological Diseases; National Key Clinical Department and Key Discipline of Neurology, No.58 Zhongshan Road 2 Guangzhou China; ^2^ Guangzhou Women and Children's Medical Center Guangzhou Medical University Guangzhou China

**Keywords:** D‐loop methylation, DNMT1, intracranial major artery stenosis/occlusion, moyamoya disease, RNF213

## Abstract

**Background:**

Mitochondrial D‐loop methylation leads to abnormal cerebral angiogenesis. This study examines its role in vascular phenotypes of moyamoya disease (MMD).

**Method:**

Blood samples from 96 intracranial artery stenosis/occlusion (ICASO) patients (35 MMD, 61 non‐MMD) and healthy controls underwent methylation analysis. D‐loop methylation levels were analyzed using chi‐square, *T*‐test, and Wilcoxon tests. Propensity score matching (PSM) adjusted for age and gender disparities. Combine clinical information with methylation data to build a diagnostic model. Further studies included the methylation levels in MMD patients carrying the RNF213 p.R4810K mutation and the methylation levels and possible mechanisms of human brain microvascular endothelial cells (hCMEC/D3) with RNF213 knockdown.

**Results:**

Healthy controls showed higher D‐loop methylation than MMD (*p* < 0.05). Post‐PSM, non‐MMD ICASO patients showed higher D‐loop methylation than MMD (*p* < 0.05). The AUC of the prediction model was 0.891 (95% CI, 0.821–0.961) after combining clinical information with methylation data. MMD patients with the RNF213 mutation exhibited reduced methylation at most sites, though not statistically significant (*p* > 0.05). RNF213 knockout in hCMEC/D3 enhanced proliferation, migration, and tube formation, while reducing apoptosis and DNMT1 expression, leading to decreased D‐loop methylation and ROS level, increased ATP production and mitochondrial membrane potential.

**Conclusion:**

There are differences in the methylation levels in the mitochondrial D‐loop region between MMD and non‐MMD ICASO. The methylation–metabolism–angiogenesis axis may represent a promising research direction for elucidating MMD pathogenesis.

## Graphical abstract

Analysis of blood samples showed that mitochondrial DNA methylation was significantly lower in MMD patients than in non‐MMD patients with intracranial artery stenosis/occlusion and healthy controls. Combined with clinical data, this difference effectively distinguished the two groups of patients, and the RNF213 gene was associated with reduced methylation levels and altered endothelial cell function.

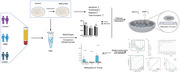

**Abbreviations**
hBMECs: human brain microvascular endothelial cells
ICASO: intracranial major artery stenosis or occlusion
MMD: moyamoya disease
RNF213: Ring finger protein 213

## 1 Introduction

Moyamoya disease (MMD) is a rare cerebrovascular disease characterized by progressive stenosis/occlusion of the terminal part of the internal carotid artery and the origins of the middle and anterior cerebral arteries, accompanied by the formation of abnormal vascular networks at the skull base. MMD is a significant contributor to intracranial major artery stenosis or occlusion (ICASO)(Kim 2016). Due to progressive vascular stenosis, MMD is characterized by recurrent ischemic attacks. The annual incidence of MMD in East Asian is estimated to be 0.5–1.5 per 100,000 people, making it one of the primary causes of stroke in children and adolescents. Although MMD and non‐MMD intracranial major artery stenosis or occlusion (non‐MMD ICASO) exhibit similar clinical manifestations, their underlying pathophysiological mechanisms differ significantly. Therefore, elucidating the specific pathophysiological mechanisms is essential for accurate diagnosis and effective treatment of MMD.

Immune dysregulation in patients with MMD was characterized by alterations in T‐cell populations, including a decrease in effector T‐cells and an increase in regulatory T‐cells (Tregs), accompanied by mitochondrial dysfunction and impaired oxidative phosphorylation capacity (Ge et al. 2024; Liu et al. 2025). DNA methyltransferase 1 (DNMT1) can translocate to mitochondria and catalyze the methylation of mitochondrial D‐loop in vascular smooth muscle cells (SMCs), thereby inhibiting mitochondrial gene expression, leading to mitochondrial functional impairment (such as respiratory chain damage) and decreased SMCs contractility (Liu et al. 2020). DNA methylation, a prevalent mechanism of epigenetic modification, modulates chromatin structure and regulates gene expression by altering nucleotide sequences. Previous studies have shown that methylation of mitochondrial DNA (mtDNA) is independent of traditional cerebrovascular risk factors and serves as a strong predictor of future cerebrovascular disease incidence (Corsi et al. 2020; Pérez‐Muñoz et al. 2021). The mitochondrial displacement loop (D‐loop) is a critical region of mtDNA governing mitochondrial gene transcription and replication (Stoccoro and Coppedè 2021). Hypermethylation of the D‐loop region has been observed in the carotid intima‐media of mice subjected to flow arrest or mechanical endothelial injury, as well as in vascular specimens from patients with carotid occlusive disease (Liu et al. 2020). Demethylation of specific sites on CpG islands within the D‐loop promoter can induce alterations in biological behaviors, including cell proliferation, apoptosis, and the cell progression (Tong et al. 2017). Currently, there is insufficient research elucidating discrepancies in mitochondrial epigenetic modifications in MMD.

RNF213 is a susceptibility gene to MMD in East Asian (Koizumi et al. 2016). The *RNF213 p.R4810K* variant increases susceptibility to both MMD and non‐MMD ICASO in individuals from Japan, Korea, and China (Miyawaki et al. 2013; Wang et al. 2018). *RNF213 p.R4810K* variant was found in 1.8% (2/110) of the normal control group and had significant associations with definite MMD (*p* < 0.0001; odds ratio, 144.0; 95% confidence interval, 26.7–775.9)(Miyawaki et al. 2013). Homozygous mutation of *RNF213p.R4810K* can lead to earlier onset of MMD and more severe clinical symptoms (Miyatake et al. 2012). ICASO carrying *RNF213 p.R4810K* mutation display imaging features of both intracranial atherosclerosis and MMD (Cheng et al. 2019). Additionally, RNF213 serves as a critical gene linking MMD and non‐MMD ICASO, as its abnormal expression can lead to the formation of pathological vascular networks. RNF213 deficiency can exacerbate pathological angiogenesis in the cortex and retina of mice and improve walking impairment and decreased blood flow caused by permanent femoral artery ligation in the hind limbs (Ito et al. 2015; Ye et al. 2023). Rnf213a knockout zebrafish display abnormal angiogenesis in intersegmental vessels and intracranial secondary vessels, along with abnormal endothelial cell morphology and lumen formation (Wen et al. 2016). However, the mechanism by which RNF213 leads to the formation of pathological vascular networks in MMD remains unclear.

Based on this, we detected and compared the methylation level of the mtDNA D‐loop region in the peripheral blood of MMD, and non‐MMD ICASO, and combined methylation data with clinical information to build a differential diagnosis model. Additionally, we constructed RNF213 knockout human brain microvascular endothelial cells (hBMECs) to further explore the regulatory effect of RNF213 on DNA methylation in the mitochondrial D‐loop region, providing new insights into the pathogenesis of MMD.

## 2 Method

### 2.1 Patients and Data Collected

Blood samples and clinic data were collected in our center from January 2016 to May 2023. Inclusion criteria for MMD patients: (1) DSA shows unilateral or bilateral internal carotid artery distal or anterior/middle cerebral artery stenosis/occlusion accompanied by reticular collateral artery formation; (2) Exclude patients with a possible diagnosis of moyamoya syndrome; (3) No recent transfusion of blood cells and platelets. Inclusion criteria for non‐MMD ICASO patients: (1) Imaging CTA/MRA/DSA shows moderate to severe stenosis of the patient's intracranial main artery without the formation of abnormal vascular network at the skull base; (2) No recent blood cell transfusion and platelets.

Trained investigators collected general clinical data of patients from the hospital's electronic medical record system. The general clinical data of the disease group mainly include: age, gender and whether there are cerebrovascular risk factors (diabetes, hypertension, hyperlipidemia, hyperuricemia, history of smoking and drinking). Fasting peripheral blood from study subjects was collected, labeled, aliquoted, and stored at −80°C until further analysis.

### 2.2 DNA Extraction and Concentration Detection

DNA was extracted using a DNA extraction kit (QIAamp DNA Blood Mini Kit, S1106, Germany). Use Nanodroop3000 (NanoDrop Technologies, Wilmington, DE, USA) to detect the concentration of the DNA obtained above, and control the concentration of DNA to > 20 ug/uL, and the total amount ≥ 400 ng.

### 2.3 DNA Methylation Detection

The MethylTarget sequencing method was used to detect the methylation level of the D‐loop region. The sample DNA was bisulfite treated using EZ DNA Methylation‐Gold Kit (ZYMO, CA, USA). Divide the D‐loop region of mtDNA (NC_012920.1) into six fragments, based on the principle of Primer F = Illumina adapter sequence 1+ specific amplification Forward primer; Primer R = Illumina adapter sequence 2+ specific amplification Reverse primer. Set six pairs of primers for multiplex PCR amplification to ensure full coverage of the D‐loop region. The amplified PCR product is diluted 10–20 times and a specific tag sequence is added. The samples were then mixed and gel‐cut, and finally high‐throughput sequencing was performed on the Illumina Hiseq (Illumina, CA, USA) platform in 2 × 150 bp paired‐end sequencing mode. The average methylation level of all CpG sites on the fragment was used as the methylation level of the fragment.

### 2.4 Primer Sequence

**Table:** 

Chr	Start	End	Length	Primer F	Primer R
MT	576	326	251	TGTGGGGGGTGTTTTTG	ACAACACTTAAACACATCTCTACCAAAC
MT	394	123	272	GAAATTTGGTTAGGTTGGTGTTAG	AATATCTATCTTTAATTCCTACCTCATCCT
MT	276	1	276	TTTGTGTGGAAAGTGGTTGTGTAGA	AATCACAAATCTATCACCCTATTAACC
MT	16,569	16,288	282	TATGTGATGTTTTATTTAAGGGGAA	TACCCACCCTTAACAATACATAATACA
MT	16,358	16,133	226	GAAGGGATTTGATTGTAATGTGTTATG	CCATAAATACTTAACCACCTATAATACATAAA
MT	16,235	16,024	212	TGTGATAGTTGAGGGTTGATTGTT	TTCTTTCATAAAAAAACAAATTTAAATACCA

### 2.5 Sequencing of Mutation Sites

Configure the PCR amplification system for the extracted DNA according to the system of 2 uL forward primer (10 umol/uL, primer sequence is as follows), 2 uL reverse primer, 5 uL 10 × Buffer, 36.4 uL enzyme‐free water, 0.6 uL PCR reaction enzyme, and 2 uL dNTP. The following cycling conditions were used for detection on LightCycler480 (Roche): 95°C for 5 min, 95°C for 20 s, 60°C for 20 s, 72°C for 20 s, and 4°C for unlimited time for PCR amplification. After the PCR product was amplified by agarose gel electrophoresis, the remaining PCR amplification product was sent to Beijing Huada for gene sequencing.

RNF213 p.R4810K detect:

RNF213‐4810F: ATTGTTACTGGGTGGTCTTCC

RNF213‐4810R: ATGCAGTGATCCTTTCGAACC

RNF213 of exon 2 detect:

SKO677‐F1: TTTCTGTCTGATTCACCCCTTGTT

SKO677‐R1: CTGGTAGAAAGAAAAGGAAGGTGG

### 2.6 Cell Culture

The hCMEC/D3 cell line used in this experiment was purchased from Zhongqiao Xinzhou. Cultivated in 95% oxygen and 5% carbon dioxide in a constant temperature incubator at 37°C. Use endothelial cell culture medium (ZQ‐1304, Shanghai Zhong Qiao Xin Zhou Biotechnology) and mix according to the proportion to prepare a complete culture medium for cell culture.

### 2.7 RNF213 Knockout

CRISPR/Cas 9 was used to construct an RNF213 knockout monoclonal hCMEC/D3 cell line by Cyagen Technology Company.

### 2.8 Real‐Time Polymerase Chain Reaction

RNA was extracted from cells using the Trizol method (A33250, Thermo Fisher Scientific, USA); RNA was reverse transcribed using Evo M‐MLV Reverse Transcription Premix Kit (AG11728, Erico, China); SYBR Green Pro was used Taq HS premixed qPCR kit II (containing Rox; AG11719, China, Eric) under the conditions of 95°C, 1 min; 95°C, 5 s; 60°C, 35 s, 40 repeated cycles of fluorescence quantitative PCR instrument (Thermo Fisher Scientific, USA) to detect CT values, and use 2‐ΔΔCT to calculate the relative expression of genes.

β‐actin‐F: 5′ CATGTACGTTGCTATCCAGGC 3′

β‐actin ‐R: 5′ CTCCTTAATGTCACGCACGAT 3′

cRNF213‐F: 5′ CACGCCAGAGCAATGTGAA 3′

cRNF213‐R: 5′ TCAAGGTTGCTGTCACTAGGCC 3′

DNMT1‐F: 5′ GCCTCAGCCTCCCAAGTAA 3′

DNMT1‐R: 5′ TCCGATTTGGCTCTTTCAG 3′

### 2.9 Western Blotting

Use according to RIPA (P0013C, BiyunTian, China); PMSF (100:1) (ST507, China, BiyunTian); phosphatase inhibitor (100:1; P1005, BiyunTian, China); the protein lysate was prepared in the proportion of EDTA (100:1; P1005, BiyunTian, China) and lysed on ice to extract proteins. Protein concentration was determined using the BCA kit (23227, Thermo Fisher Scientific, USA) and diluted with 5 × loading buffer. The gel was prepared by PAGE rapid gel preparation kit (PG111, Yase, China). Electrophoresis and membrane transfer were carried out by vertical high‐pressure electrophoresis system. The cells were blocked with 5% milk for 1 h. Antibodies were diluted with the use of a primary antibody diluent: DNMT: 1:1:1000 (5032T, Cell Signaling Technology, USA); GAPDH: 1:3000 (Cell Signaling Technology, USA); GAPDH: 1:1:1000; the primary antibody was incubated at 4°C overnight. The secondary Anti‐rabbit IgG HRP‐linked Antibody (1:10,000) was incubated at room temperature. The exposure solution (P10100, BiyunTian, China) was prepared and imaged by Western blot imaging instrument and ImageLab software.

### 2.10 Cell Counts

Digest the cells with 0.05% trypsin, centrifuge and resuspend the cells, and count the cells using a red blood cell counting plate. Count the number of cells in the square grid and calculate the number of cells per mL according to the following formula:

*X*/80 × 400 × 10 × 1000 = 1 dm^3^ (1 mL)

*X* is the number of cells in five medium squares (i.e., 80 small squares)

400 is the number of small squares contained in a large square (1 mm^2^)

10 is the actual height of the cover glass and counting plate is 1/10 mm

1000:1 mm^3^ = 1 uL = 10^−3^ mL

### 2.11 Transwell

After counting, cells were diluted by adding serum‐free medium at a density of 3 × 10^4^ cells/100 uL; add 500 uL of complete endothelial cell culture medium to the bottom of the 24‐well plate, and slowly insert it into the Transwell chamber to avoid the formation of a gap between the bottom of the chamber and the culture medium. Air bubbles; add 100 uL of resuspended cell solution into each chamber and place it in a 37°C cell culture incubator. After 12 h, the cells were fixed with 4% paraformaldehyde for 20 min and stained with crystal violet for 5 min at room temperature. A fully automated inverted fluorescence microscope captures images under 20 × magnification and records the number of cells that migrate to the lower surface.

### 2.12 Tube Experiment

The pipette tip and angioplasty slide were placed on ice to precool, and Matrigel (356234, Corning, USA) was placed on ice to thaw at low temperature. Add 10 uL of Matrigel to each culture well and let it stand on an ice brick for about 10 min to make the glue surface flat; place it in a 37°C cell culture incubator for 2 h to solidify the Matrigel. Count the cells using a cell counting plate, dilute the cell density to 8 × 10^3^/40 uL using serum‐free medium, and add 40 uL of the diluted and resuspended cell solution to each culture medium. After the spotting is completed, culture it in a 37°C cell culture incubator for 12 h; observe with an inverted microscope with a 20x objective lens, and use ImageJ to count the number of tubes, nodes, and tube area of the cells.

### 2.13 CCK‐8

The cells obtained after digestion and counting were set at a density of 3 × 10^3^/100 uL. Each group was set with three multiple wells and four time points of 0 h, 24 h, 48 h, and 72 h (each time point was inoculated in a different 96‐well plate. We inoculate the cells into a 96‐well plate, record the inoculation time, and place the cells in a 37°C incubator. After 2 h of sinking to the bottom, take the cell plate at the 0 h time point. After adding 10 uL of CCK‐8 reagent to each well, record the sample time at point CCK‐8. This time is recorded as 0 h where the growth curve is located. Place the well plate in a 37°C incubator. Incubate for 2 h, and use a microplate reader to detect the absorbance at a wavelength of 450 nm. Detect the absorbance at 24 h, 48 h, and 72 h; draw the absorbance at 450 nm detected at each time point on a line graph, and calculate the proliferation trends of different groups of cells at different time points.

### 2.14 Annexin V‐FITC

The cells obtained after digestion were seeded on a six‐well plate at a ratio of 1:4 and cultured for 36 h. Remove old culture medium and wash twice with PBS. Add 0.5 mL of 0.05% trypsin to each well to digest the cells; incubate at room temperature for 2 min until the cells can fall off by gently pipetting. Aspirate the trypsin cell digestion solution and add the old culture medium collected in advance to terminate digestion. Collect the cells and transfer them to a centrifuge tube, centrifuge at 1000 × *g* for 5 min, discard the supernatant, collect the cells, gently resuspend the cells in PBS and count. Take 10 × 10^4^ resuspended cells from each tube into a 1.5 mL centrifuge tube, centrifuge at 1000 × *g* for 5 min, and discard the supernatant. Add the stain according to the instructions and incubate at room temperature in the dark for 20 min. During this period, the cells are resuspended 2–3 times, and then placed in an ice bath in aluminum foil to avoid light. Use flow cytometry to detect: Annexin V‐FITC is green fluorescence, and propidium iodide is red fluorescence.

### 2.15 Mitochondrial Membrane Potential Detection

The cells obtained after digestion were seeded on a six‐well plate at a ratio of 1:4 and cultured for 36 h. Aspirate off the old culture medium, wash twice with PBS, and then add 1 mL of complete cell culture medium to each well. Add 1 mL of JC‐1 staining working solution to each well (preparation ratio: JC‐1 (200 ×): JC‐1 staining buffer = 1:200), mix thoroughly and place in a 37°C cell culture incubator for 20 min. Aspirate the supernatant, wash twice with JC‐1 staining buffer, transfer the digested cells to a centrifuge tube, centrifuge to remove the supernatant, and resuspend in 2 mL of cell culture medium. Observation on the flow cytometer: When JC‐1 is a monomer, the excitation light wavelength can be set to 490 nm, and the emission light wavelength can be set to 530 nm; when JC‐1 polymer is detected, the excitation light wavelength can be set to 525 nm, and the emission light wavelength can be set to 525 nm. Set to 590 nm.

### 2.16 ATP Production Detection

Aspirate the culture medium and lyse the cells by adding 200 µL of lysis buffer to each well of a six‐well plate. Centrifuge at 12,000 × *g* for 5 min at 4 plate. Centrifuge at 12,f a se the cells by adding 200 th can ATP standard solution with ATP Assay Lysis Buffer to the appropriate concentration gradient. Prepare the appropriate amount of ATP Assay Working Solution, using 100 µL of ATP Assay Working Solution for each sample or standard. Thaw the reagents on ice. Take an appropriate amount of ATP Assay Reagent and dilute it 1:9 with ATP Assay Reagent Diluent. Add 100 µL of ATP Assay Working Solution to the assay wells or tubes. Incubate at room temperature for 3–5 min to deplete background ATP and reduce background. Add 20 µL of sample or standard to the assay wells or tubes and quickly mix using a micropipette. After at least 2 s, measure the RLU using a chemiluminescence analyzer (GloMax 20).

### 2.17 ROS Level Detection

Digest the cells with 0.25% trypsin and resuspend them in DCFH‐DA. Incubate in a 37°C cell culture incubator for 20 min. Invert and mix every 3–5 min to ensure thorough contact between the probe and the cells. Wash the cells three times with PBS to remove any remaining DCFH‐DA. Flow cytometry (BD Celesta) was used to measure fluorescence before and after stimulation, using an excitation wavelength of 488 nm and an emission wavelength of 525 nm. Fluorescence intensity was measured in real time or at time points.

### 2.18 Statistical Analysis

Statistically describe and analyze the methylation levels and clinical data of different groups of people. Categorical variables are described as percentages, numerical variables are described as mean or median, and chi‐square test, *T*‐test and Wilcoxon are used for comparison between groups. When there were statistical differences in baseline data between groups, the propensity score matching (PSM; caliper value: 0.03) was used to equalize the differences between groups. Use Spearman for correlation analysis.

Least absolute shrinkage and selection operator LASSO regression were used to screen data dimensions and predictors, and establish MMD and non‐MMD ICASO differential diagnosis prediction models. The discriminative ability of the model is evaluated by the receiver operating characteristic curve (AUC). The discrimination of the model is evaluated by the *C* index. Assess the calibration of the predictive model using the Hosmer–Lemeshow goodness of fit. The decision analysis curve was used to evaluate the clinical predictive value of the model. Use bootstrap resampling to internally verify the model and evaluate the accuracy of model predictions.

This study uses stats [4.2.1], ggplot [3.3.6], glmnet [4.1.7], rms [6.4.0], ResourceSelection [0.3–5], rmda [1.6] of R (4.2.1), IBM SPSS 25.0 (SPSS Inc, Armonk, NY, USA) was used to perform propensity matching analysis on the model with baseline mismatch. *p* < 0.05 indicates a statistical difference. All examinations are two‐sided unless otherwise stated.

## 3 Results

### 3.1 Comparison of Methylation Levels Between Healthy Controls and MMD

**FIGURE 1:**
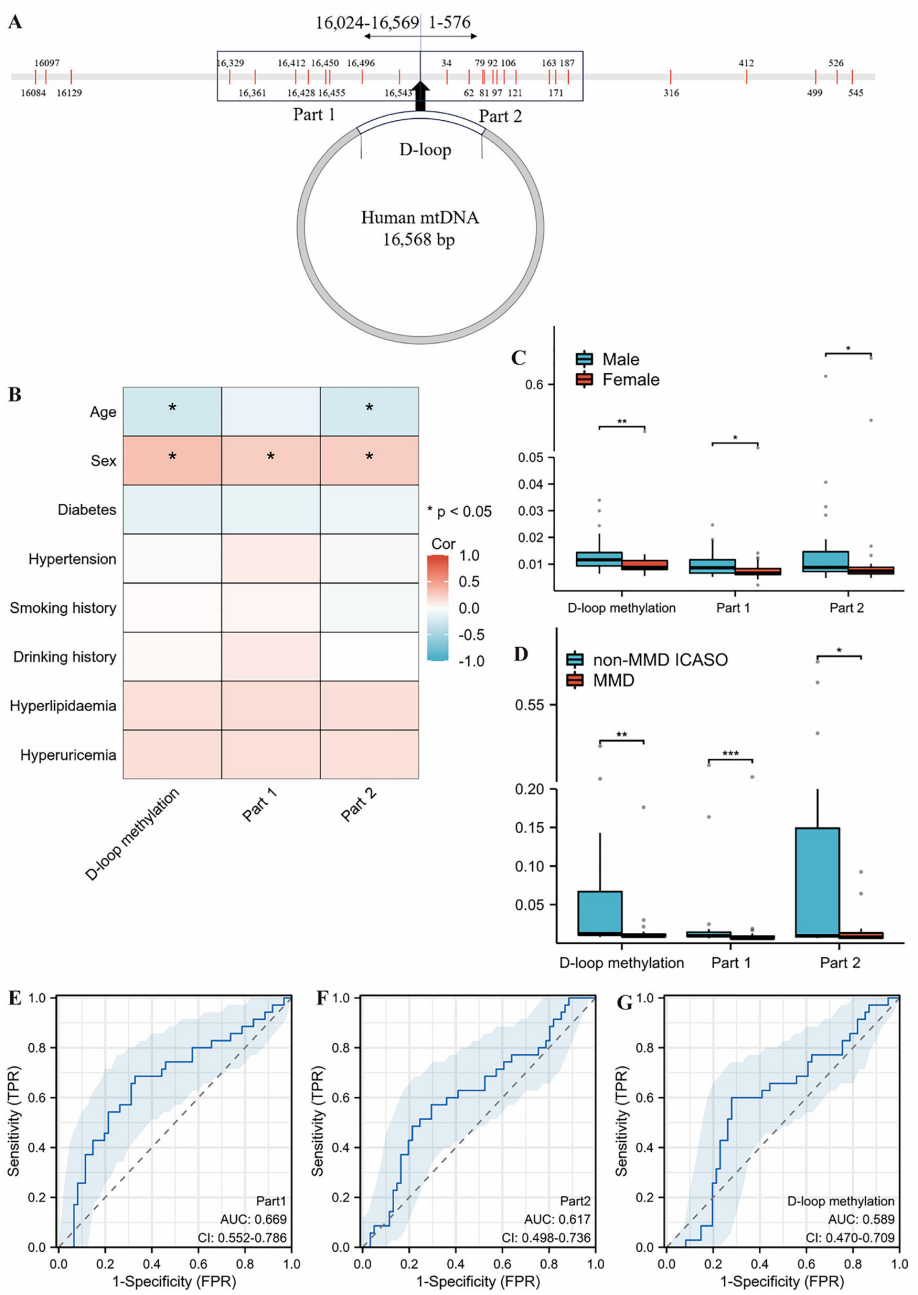
Comparison of methylation levels between disease groups. (A) Schematic diagram of the distribution of methylation sites in the D‐loop region of mtDNA: Part 1 covers the following sites: 16,329, 16,361, 16,412, 16,428, 16,450, 16,455, 16,496, 16,453; Part 2 covers the following sites: 34, 62, 79, 81, 92, 97, 106, 121, 163, 171, 187. (B) Heat map of single‐factor correlation analysis of factors affecting methylation levels in the ICASO group: Age and gender showed correlation with methylation levels in the ICASO group. (C) Comparison of methylation levels of different genders in the ICASO group. Male patients have higher methylation levels than female patients. (D) Comparison of overall and fragment methylation between non‐MMD ICASO and MMD groups; non‐MMD ICASO patients have higher methylation modification levels in Part 1, Part 2 and overall methylation levels than MMD patients. (E) Plot of AUC for Part 1: AUC = 0.669 (95% CI, 0.552–0.786). (F) Plot of AUC for Part 2: Part 2 AUC = 0.617 (95% CI, 498–0.736). (G) Plot of AUC for D‐loop methylation: AUC = 0.589 (95% CI, 0.470–0.709). ICASO, intracranial major artery stenosis/occlusion; MMD, moyamoya disease. *p < 0.05; **p < 0.01; ***p < 0.001.

The sequences of the D‐loop region were 1–576 and 16,024–16,569 (NC_012920.1). A total of 27 CpG sites were identified as being susceptible to methylation modifications (Figure 1A). Most of the methylation sites were highly concentrated in two regions, and other sites were scattered. Based on the distribution of methylation sites, the first part (Part 1) of the study included the region from 0 to 200 (11 CpG), while the second part (Part 2) covered the region from 16,300 to 16,543 (8 CpG).

Methylation levels in the D‐loop region were compared between 35 MMD patients and 20 healthy controls. At position 34, 81, 545, 16,129, and Part 1 the methylation level of the healthy control was higher than that of the MMD (*p* < 0.05; Supporting Information Table 1). There was also no abnormality in methylation levels among MMD groups with different initial symptoms in most sites (Supporting Information Table 2).

### 3.2 Comparison of Methylation Levels Between MMD and Non‐MMD ICASO

This study included a total of 96 ICASO patients, consisting of 61 non‐MMD ICASO patients and 35 patients with MMD. There was a significant statistical difference in the age, diabetes, hypertension, smoking history, drinking history, and hyperlipidemia of the two groups of patients (*p* < 0.05; Table 1). After excluding four MMD patients due to incomplete clinical data, Spearman's single‐factor correlation analysis was performed to assess the impact of baseline factors on both overall and segmental D‐loop methylation (Figure 1B and Supporting Information Figure 1). In all the ICASO patients, age was negatively correlated with overall and Part 2 fragment methylation levels (*p* < 0.05), and male ICASO patients had higher methylation levels (Figure 1C). A similar trend was observed in the non‐MMD ICASO group (Supporting Information Table 3 and Supporting Information Figure 1). However, no significant correlation was found between age and overall as well as fragment methylation levels in the MMD group (Supporting Information Figure 1).

**TABLE 1:** Baseline comparison before and after MMD and non‐MMD ICASO matching.

**Characteristic**	**Before PSM**			**After PSM‐1**		
	**Non‐MMD ICASO**	**MMD**	***p***	**Non‐MMD ICASO**	**MMD**	***p***
*n*	61	35		21	21	
Age, median (IQR)	55 (51, 58)	45 (34.5, 51)	< 0.001***	49 (46, 54)	50 (45, 52)	0.801
Male, *n* (%)	42 (68.9%)	21 (60%)	0.379	12 (57.1%)	14 (66.7%)	0.525
Diabetes, *n* (%)	21 (34.4%)	2 (6.5%)	0.003**	5 (23.8%)	2 (9.5%)	0.408
Hypertension, *n* (%)	42 (68.9%)	8 (25.8%)	< 0.001***	13 (61.9%)	7 (33.3%)	0.064
Smoking history, *n* (%)	30 (49.2%)	8 (25.8%)	0.031*	8 (38.1%)	5 (23.8%)	0.317
Drinking history, *n* (%)	25 (41%)	4 (12.9%)	0.006**	7 (33.3%)	4 (19%)	0.292
Hyperlipidemia, *n* (%)	23 (37.7%)	4 (12.9%)	0.014*	11 (52.4%)	3 (14.3%)	0.009*
Hyperuricemia, *n* (%)	7 (11.5%)	1 (3.2%)	0.349	3 (14.3%)	1 (4.8%)	0.599

There is a significant statistical difference in the baseline levels of the two groups of patients (*p* < 0.05). After excluding four MMD patients with incomplete clinical data, PSM was employed to balance gender and age distribution between the two groups.

Abbreviations: ICASO, intracranial major artery stenosis/occlusion; IQR, interquartile range; MMD, moyamoya disease; PSM, propensity score matching; *p < 0.05; **p < 0.01; ***p < 0.001.

After excluding four MMD patients with incomplete clinical data, PSM was employed to balance gender and age distribution between the two groups. All baseline characteristics excepted for hyperlipidemia distribution were successfully balanced (Table 1 and Supporting Information Figure 2). The non‐MMD ICASO patients had higher methylation levels at 34, 62, 79, 81, 92, 97, 106, 121, 499, 545, 16,084, 16,329, 16,361, 16,428, 16,450, 16,496 (*p* < 0.05; Supporting Information Table 4). Among them, eight sites were in Part 1 and five sites were in Part 2. The methylation levels of Part 1 and Part 2 of the two groups of patients were further analyzed independently, and the results were consistent with the above results (*p* < 0.05; Figure 1D). Methylation levels had a certain ability to distinguish between MMD and non‐MMD ICASO, among which Part 1 AUC = 0.669 (95% CI, 0.552–0.786); Part 2 AUC = 0.617 (95% CI, 498–0.736); D‐loop methylation AUC = 0.589 (95% CI, 0.470–0.709; Figure 1E, G). DeLong's test was used to test the discrimination ability among the three. The discrimination ability of Part 1 was better than that of the overall methylation level (*p* < 0.05).

### 3.3 Clinical Differential Diagnosis Prediction Model

**FIGURE 2:**
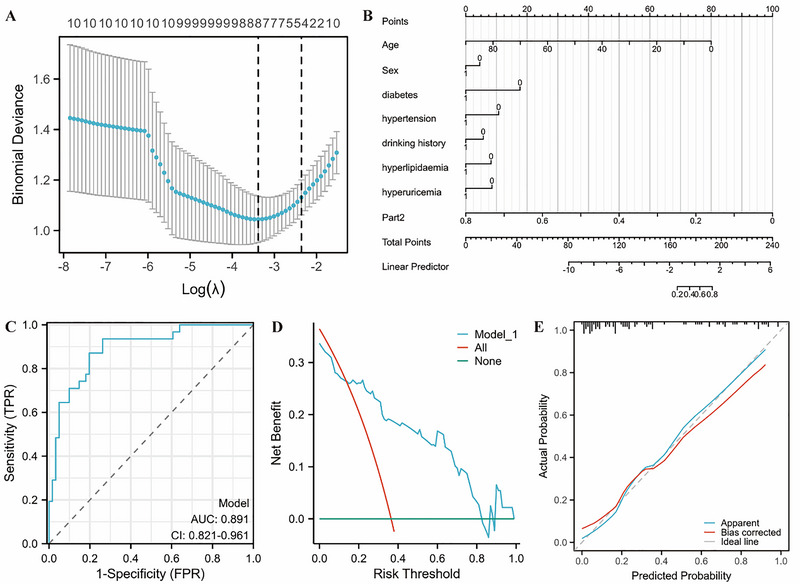
Model construction and validation. (A) Diagnostic LASSO coefficient screening chart: Predictive factors were selected using LASSO regression analysis and 10‐fold cross‐validation. Tuning parameter (lambda) selection for bias in LASSO review based on the minimum criterion (left dashed line) and 1‐SE criterion (right dashed line), where there are a total of eight variables shown on the left dashed line. (B) Diagnostic nomogram: Points: indicates the single score corresponding to each predictor variable under different values; Total points: indicates the corresponding score after all variables take values. The individual scores corresponding to each variable in patients with large artery stenosis are then added together to obtain the total score, and the probability of the patient suffering from moyamoya disease is found based on the minimum rule. (C) Diagnostic ROC curve: ICASO and MMD differential diagnosis prediction model nomogram. (D) Diagnostic model calibration curve: the abscissa is the survival probability predicted by the model; the ordinate is the actual observed survival probability. The Apparent curve represents the prediction curve, the bias‐corrected curve represents the calibration curve, and the ideal curve represents the ideal curve; the closer it is to the diagonal. The line indicates a better fit. Hosmer–Lemeshow goodness of fit produced p = 0.3125, indicating that the model fits well. (E) Diagnostic DCA chart: The abscissa is the risk probability threshold, the ordinate represents the net rate of return, and the curve represents the change of the net rate of return of each variable (each model) with the high risk probability threshold, where All: represents all everyone intervenes, None: means no intervention at all, and the net benefit is always 0; as the high‐risk probability threshold increases, the net benefit of intervention based on the model results will decrease. If the model is stably higher than the reference line within the risk probability threshold interval of 0.2–0.8, it means that the model is better. ICASO, intracranial major artery stenosis/occlusion; LASSO, least absolute shrinkage and selection operator; MMD, moyamoya disease; SE, standard error. *p < 0.05; **p < 0.01; ***p < 0.001.

The LASSO regression method was used to select eight variables from the collected clinical data (eight variables) and methylation values (three variables) to build the ICASO clinical differential diagnosis prediction model of MMD and non‐MMD. The selected variables comprise: age, gender, diabetes, hypertension, drinking history, hyperlipidemia, hyperuricemia, and methylation of Part 2 (Figure 2A). The above model is presented in the form of a nomogram (Figure 2B).

The AUC of the prediction model was 0.891 (95% CI, 0.821–0.961; Figure 2C). Internal validation using bootstrap by10‐fold cross‐validation (resampling = 1000) yielded an accuracy of 0.784 and a model stability kappa value of 0.480, suggesting moderate consistency of the model. The *C* index was used to assess the discriminative ability of the model. In this study, the *C* index was 0.891 (95% CI, 0.821–0.961), indicating moderate accuracy of the model. Use Hosmer–Lemeshow goodness of fit to conduct a goodness‐of‐fit test (Figure 2D), resulting in *p* = 0.313 > 0.05, indicating good fit between the predicted and observed values. The clinical decision curve assessed the model's clinical utility (Figure 2E,C), demonstrating consistent superiority of the model over the reference line within the risk probability threshold interval of 0.2–0.8.

**FIGURE 3:**
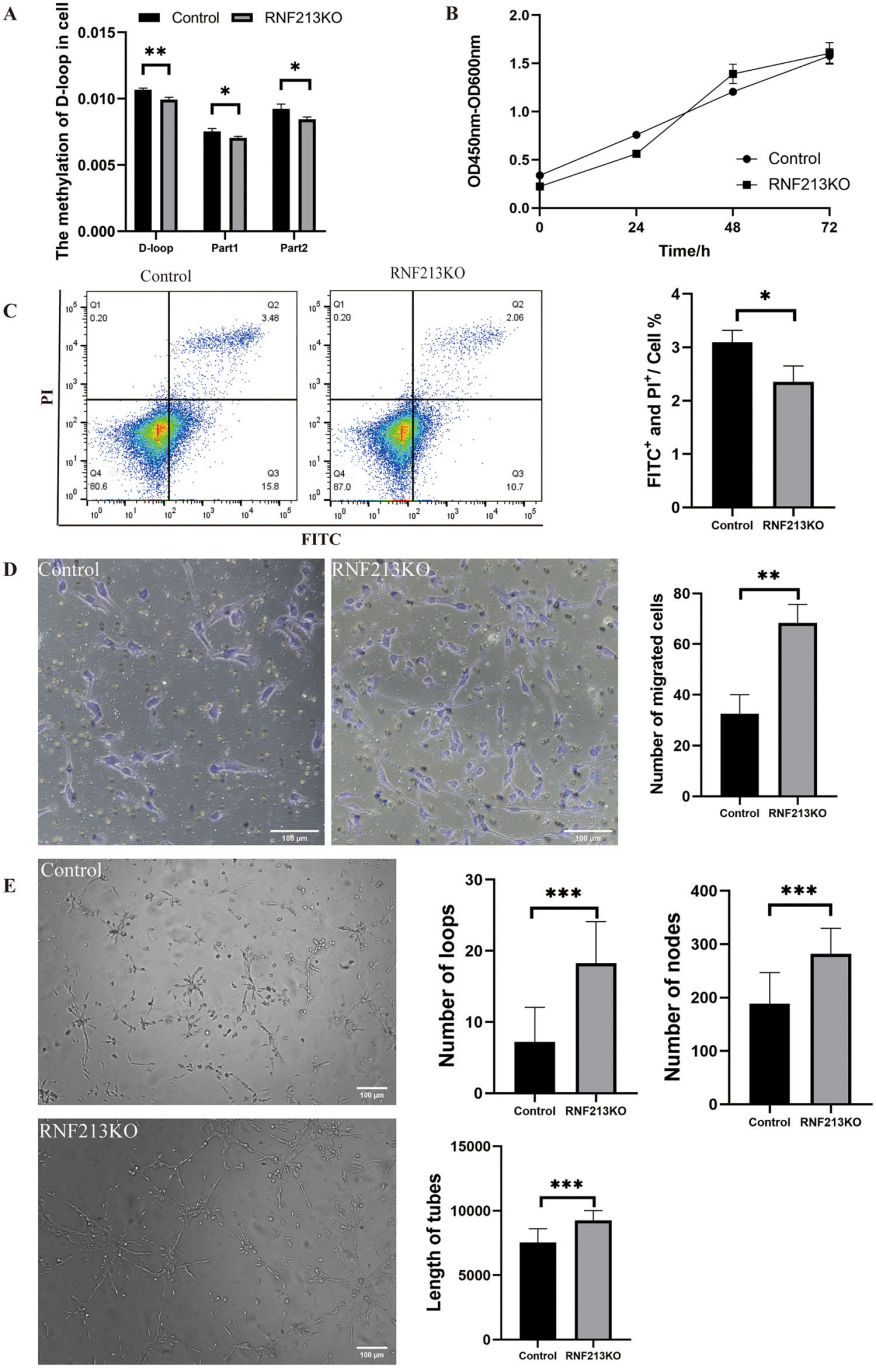
Effects of RNF213 knockout on cells. (A) D‐loop changes after RNF213KO: RNF213KO leads to a significant decrease in overall and fragment methyl levels in the mitochondrial D‐loop region. (B) RNF213KO cell proliferation changes: RNF213 knockout can lead to cell proliferation between 24 and 48 h. The ability is enhanced and reaches the upper limit of growth by 72 h. (C) Apoptotic changes in RNF213KO cells: RNF213KO can show the apoptosis ratio of scanned cells. (D) Changes in the vertical migration ability of RNF213KO cells: RNF213KO can increase the ability of cells to migrate vertically. (E) Changes in the tube‐forming ability of RNF213KO cells: The tube‐forming ability of RNF213KO cells was significantly enhanced. Compared with the control group, the tube length, number of tube‐forming nodes, and number of tube‐forming rings of RNF213KO cells were significantly increase. *p < 0.05; **p < 0.01; ***p < 0.001.

### 3.4 The Role of RNF213 on Mitochondrial Methylation

#### 3.4.1 Mitochondrial Methylation Levels in MMD Patients With and Without *RNF213 p.R4810K* Mutation

We found four MMD patients carrying the *RNF213 p.R4810K* mutation, resulting in a mutation rate of 11.43%. Three MMD patients matched for age and gender, who lacked the *RNF213 p.R4810K* mutation, were selected to compare their mitochondrial methylation levels with those carrying the mutation (Supporting Information Table 5). Patients with this mutation exhibited lower methylation levels at most sites compared to nonmutation carriers, although this difference was not statistically significant (*p* > 0.05).

#### 3.4.2 Impact of RNF213 Knockout on Mitochondrial Methylation

Previous studies have demonstrated that RNF213 is a functional nonsecreted cytoplasmic protein expressed in brain endothelial cells (Kim 2016). The researchers employed CRISR/Cas 9 knocked out RNF213 (Supporting Information Figure 3). The test indicated that knockout of RNF213 can lead to a significant decrease in the methylation level of the D‐loop region (Figure 3A). Additionally, RNF213 knockout resulted in enhanced cell proliferation between 24 and 48 h, reaching the growth limit by 72 h (Figure 3B). Conversely, the apoptosis rate of RNF213 knockout cells decreased (Figure 3C), while their vertical migration and tube formation abilities were significantly enhanced (Figure 3D,E). In summary, RNF213 knockout resulted in a hypomethylated state of the mitochondrial D‐loop and activated cellular functionality.

#### 3.4.3 RNF213 Regulates the Methylation Level of Mitochondrial D‐Loop Region via DNMT1

**FIGURE 4:**
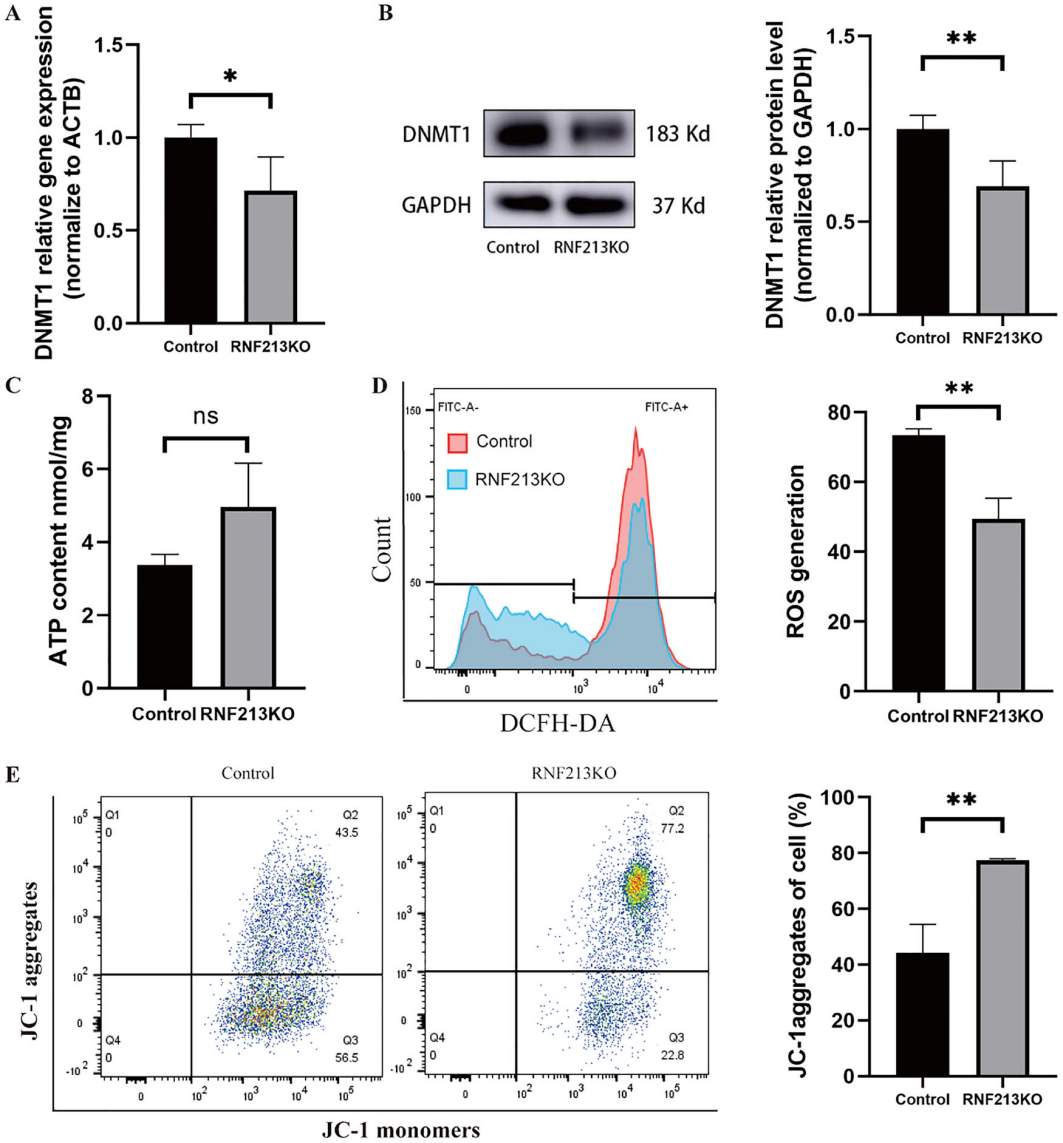
The mechanism by which RNF213 causes cellular changes. (A) Changes in DNMT1 transcription: Compared with the control group, RNF213KO can lead to a decrease in the mRNA produced by DNMT1 transcription. (B) Changes in DNMT1 translation: Compared with the control group, RNF213KO can lead to a decrease in the protein produced by DNMT1 translation. (C) Changes in mitochondrial membrane potential in RNF213KO: Compared with the control, RNF213KO can lead to an increase in mitochondrial membrane potential levels. *p < 0.05; **p < 0.01.

The DNA methyltransferase (DNMT) family encompasses conserved DNA modification enzymes, primarily DNMT1, DNMT3A, and DNMT3B, which are crucial for epigenetic regulation (Kim et al. 2013). Among them, DNMT1 was one of the three proteins currently known to target and regulate mtDNA methylation levels (Kim et al. 2013; Ryoo et al. 2014; Yu et al. 2015). After RNF213 knockout, DNMT1 expression was reduced (Figure 4A,B). ATP production was enhanced in cells after RNF213 knockout, but there was no statistical difference compared to the control group. However, mitochondrial membrane potential increased and ROS levels decreased after RNF213 knockout, indicating enhanced mitochondrial function (Figure 4C,D,E).

## 4 Discussion

D‐loop methylation levels in the entire region, as well as in Parts 1 and 2, were higher in non‐MMD ICASO patients compared to those with MMD. Age and gender influenced D‐loop region methylation levels in non‐MMD ICASO patients. The AUC of the prediction model was 0.891 (95% CI, 0.821–0.961) after combining clinical information with methylation data. MMD patients with the RNF213 mutation exhibited reduced methylation at most sites, though not statistically significant (*p* > 0.05). RNF213 knockout in hCMEC/D3 enhanced proliferation, migration, and tube formation, while reducing apoptosis and DNMT1 expression, leading to decreased D‐loop methylation and ROS level, increased ATP production and mitochondrial membrane potential. To our knowledge, this study is the first to focus on the association between D‐loop methylation and RNF213.

DNA methylation is a well‐explored epigenetic process that alters chromatin structure and gene expression by modifying nucleotide structure (Coppedè and Stoccoro 2019). DNA methylation levels can serve as diagnostic markers in diseases such as Alzheimer's disease (Silva et al. 2022), cerebral small vessel disease (Yang et al. 2023), ischemic stroke (Qin et al. 2019), and so on. It is difficult to distinguish non‐MMD ICASO from MMD in the early stage, especially when there is no moyamoya angiogenesis. In addition, ICASO caused by atherosclerosis tends to be younger, which increases the difficulty of diagnosing and distinguishing MMD. This study found that the overall and part D‐loop methylation level of non‐MMD ICASO was higher than that of the MMD group. Given the limited discriminatory power of D‐loop methylation (AUC = 0.589–0.669), the AUC of the prediction model was 0.891 (95% CI, 0.821–0.961) after combining clinical information with methylation data. Direct comparison with imaging‐based gold standards (such as DSA and MRA) is essential to validate the clinical applicability of our diagnostic model, particularly for early‐stage MMD detection. Therefore, further validation is required to assess its biomarker potential and liquid biopsy utility.

This study found that gender and age can affect the methylation level of the D‐loop region in the ICASO group. Overall methylation levels in male patients within the ICASO group were higher than those of female patients, consistent with findings from studies on cerebral autosomal dominant arteriopathy with subcortical infarction and leukoencephalopathy, as well as amyotrophic lateral sclerosis (Stoccoro et al. 2020; Zhang et al. 2022). In the ICASO and non‐MMD ICASO group, age was negatively correlated with overall and Part 2 fragment methylation levels (*p* < 0.05). Previous studies have demonstrated that the methylation of some gene loci can become hypermethylated or hypomethylated with age (Unnikrishnan et al. 2019). Decrease in D‐loop methylation levels with age in individuals with amyotrophic lateral sclerosis, particularly those over the age of 60 (Unnikrishnan et al. 2019). Compared with proliferating endothelial cells, the D‐loop of aging endothelial cells is more susceptible to demethylation. However, methylation levels in MMD are not influenced by age. The distinct age distributions between MMD and non‐MMD ICASO likely contribute to differing correlations between D‐loop region methylation and age in the respective groups. Onset age distribution for MMD exhibits a bimodal pattern, occurring between 10 and 19 years old in children and approximately 40–49 years old in adults (Zhang et al. 2022). The mean age of onset of ischemic symptoms and bleeding symptoms of ICASO in Chinese population was (65.9 ± 13.0) years and (64.5 ± 11.524) years, respectively (Sun et al. 2020). In this study, the median age of the non‐MMD ICASO group was 55 years, which was greater than the 45 years of the MMD group (*p* < 0.001). Non‐MMD ICASO patients, particularly atherosclerotic ICASO, tend to be older and exhibit higher rates of diabetes, hypertension, dyslipidemia, and smoking compared to those with MMD patients (Jeon et al. 2014; Lei et al. 2014). The mechanism by which age and gender affect the methylation level of the D‐loop region needs further study.

The *RNF213 p.R4810K* variant is most strongly associated with MMD, but the penetrance is lower than 1% (Ihara et al. 2022). The number of patients with MMD, which was estimated at approximately one per 300 carriers of *RNF213 p.R4810K* in east Asian (Liu et al. 2012). MMD patients carrying *RNF213 p.R4810K* exhibited lower methylation levels at most sites in the D‐loop region compared to noncarriers. Currently, it is indicated that D‐loop is associated with alterations in mtDNA gene transcription and mitochondrial replication (Stoccoro and Coppedè 2021). Methylation of mtDNA induced by the DNMT1 enzyme in conjunction with the D‐loop can regulate the expression of mtDNA genes (Shock et al. 2011). Previous studies have shown that transplantation of DNMT1‐deficient mitochondria can restore the constrictive function of ligated vasculature (Liu et al. 2020). DNMT1‐mediated D‐loop hypermethylation in the intima‐media of ligated mice and human tissue from arterial stenosis–occlusive disease can inhibit mitochondrial gene expression, leading to mitochondrial respiratory defects and impaired contractile function (Liu et al. 2020). The study results indicated that RNF213 knockout reduce the expression of DNMT1, leading to decreased methylation levels in the mitochondrial D‐loop region, increased mitochondrial membrane potential levels, and enhanced endothelial cell function. In contrast to atherosclerosis‐associated intracranial arterial stenosis/occlusion (AS‐ICASO), MMD exhibits downregulated expression of genes associated with mitochondrial function and oxidative phosphorylation in the intracranial arterial gene expression profile (Xu et al. 2022). Variations exist in the levels of metabolites such as glutamine, taurine, and glucose in the cerebrospinal fluid of MMD and non‐MMD ICASO (Jeon et al. 2015). The combination of DNMT1 inhibitors and histone deacetylase inhibitors can synergistically activate methylation‐silenced genes (Jakobsen et al. 2025). In liver cancer, combined inhibition of mTOR and DNMT1 significantly suppresses tumor growth by downregulating DNMT1 translation (Chen et al. 2023). DNMT1 knockout suppresses chemoresistance and metastasis in breast cancer by upregulating miR‐497 (Liu et al. 2022). A similar mechanism was also observed in oral squamous cell carcinoma, where DNMT1 inhibition significantly reduced tumorigenesis (Liu et al. 2024). We first to focus on the association between D‐loop methylation and RNF213.Therefore, epigenetic modifications, specifically methylation regulated by DNMT1, may play a crucial role in RNF213‐induced MMD‐specific neovascularization.

This study found that the pathogenic mechanisms of MMD and non‐MMD ICASO may differ at the mitochondrial epigenetic level. However, this study still has many shortcomings (Chen et al. 2023): The blood sample source is single and only includes southern Chinese populations. The results may not be applicable to northern China or other ethnic groups (Cheng et al. 2019). Because this study is a single‐center study with a small sample size, there is currently insufficient data for external validation, and it needs to be verified in a larger multicenter cohort (Coppedè and Stoccoro 2019). The current research results only partially elucidate the functional consequences of RNF213 deficiency, and the intermediate links connecting D‐loop methylation, mitochondrial dysfunction and angiogenesis disorders are still incomplete. Future studies should further clarify the regulatory mechanisms of RNF213 on mitochondrial function. DNMT1 rescue experiments should be conducted to establish a clearer causal relationship. The methylation–metabolism–angiogenesis axis may represent a promising research direction for elucidating MMD pathogenesis.

## 5 Conclusion

There are differences in the methylation levels in the mitochondrial D‐loop region between MMD and non‐MMD ICASO. The methylation–metabolism–angiogenesis axis may represent a promising research direction for elucidating MMD pathogenesis.

## Author Contributions

Yuting Luo and Heng Ye contributed equally to this paper. Shaoqing Wu and Xunsha Sun designed and directed this study. Yuting Luo designed to detect the D‐loop methylation and mutations of RNF213. After that Heng Ye and Chutong Guo collected the data of patients. Yuting Luo and Heng Ye analyzed this data. Yuting Luo drafted the manuscript. Shaoqing Wu, Xunsha Sun, Chutong Guo, and Heng Ye provided some constructive comments to help revise the manuscript. All the authors have approved the final version of the manuscript.

## Funding

This study was supported by grants from the National Natural Science Foundation of China (82271333, 82071292), Kelin New Talent Program (R08016), Science and Technology Projects in Guangzhou (202201020652), Guangdong Provincial Clinical Research Center for Neurological Diseases (2020B1111170002), Guangdong Province International Cooperation Base for Early Intervention and Functional Rehabilitation of Neurological Diseases (2020A0505020004), Guangzhou Major Difficult and Rare Diseases Project (2024MDRD02), Guangdong Provincial Engineering Center for Major Neurological Disease Treatment, Guangdong Provincial Translational Medicine Innovation Platform for Diagnosis and Treatment of Major Neurological Disease, Guangzhou Clinical Research and Translational Center for Major Neurological Diseases.

## Ethics Statement

This study complied with the Ethical Declaration of Helsinki. Ethical approval was carried out by the Clinical Research and Experimental Animal Ethics Committee of the First Hospital of Sun Yat‐sen University [2022]001. All participants agreed to participate in this study.

## Consent

All the authors have approved the final version of the manuscript for publication.

## Conflicts of Interest

The authors declare no conflicts of interest.

## Supporting information



Supporting Information Figures: brb371042‐sup‐0001‐figuresS1‐S3.docx

Supporting Information Tables: brb371042‐sup‐0001‐tablesS1‐S5.docx

## Data Availability

The datasets generated and/or analyzed during the current study are not publicly available, but are available from the corresponding author upon reasonable request. The materials used in this study are available from the corresponding author upon reasonable request. Requests for data access can be directed to Xunsha Sun.
