# Supplementary material for: Study on Abnormal Angiogenesis in Moyamoya Disease via Mitochondrial D‐Loop Methylation
Source: Brain Behav. 2025 Nov 21;15(11):e71042. doi: 10.1002/brb3.71042 (PMC12639187; doi:10.1002/brb3.71042)
Supplement: Supplementary file 1 — Supporting Information Figures: brb371042‐sup‐0001‐figuresS1‐S3.docx [file BRB3-15-e71042-s001.docx]

Supplementary Figure 1: Correlation analysis of methylation influencing factors in the MMD and non-MMD ICASO groups


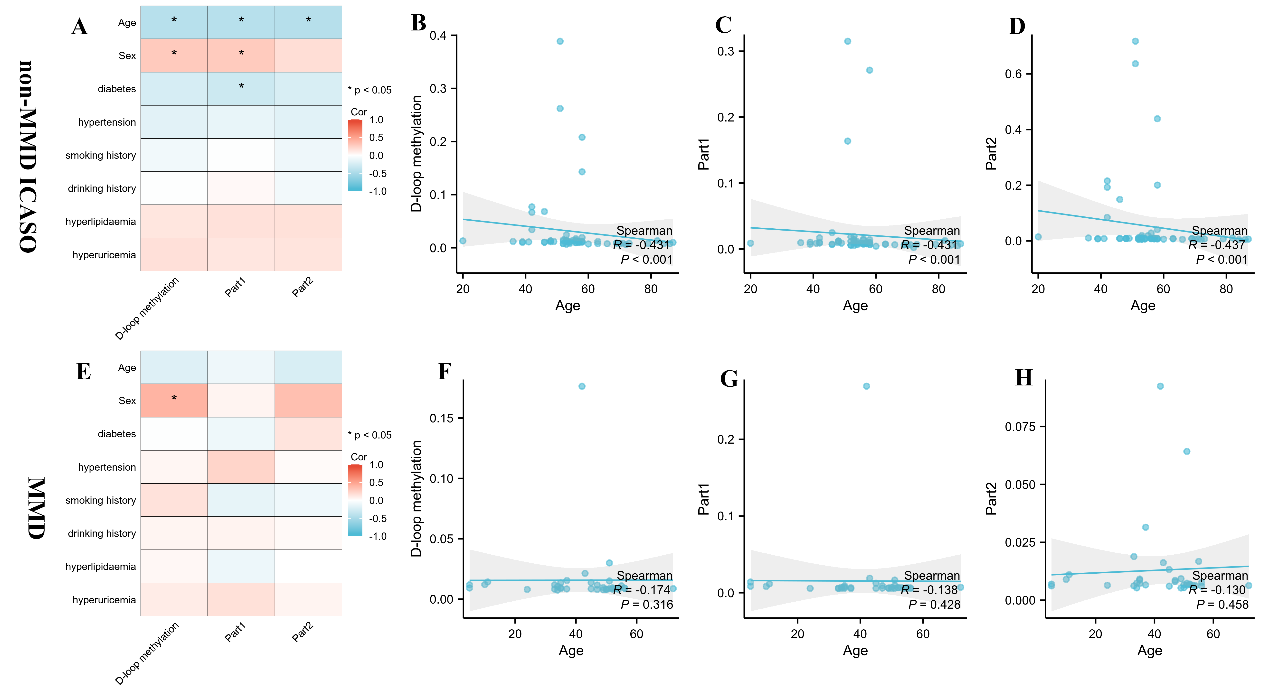


Figure A: Single-factor correlation analysis heat map of factors affecting methylation levels in the non-MMD ICASO group. Age, gender, and diabetes were correlated with the methylation level analysis of the D-loop region. B-D: Scatter plots of the correlation between age and methylation level distribution of the whole and fragments of the D-loop region in the non-MMD ICASO group. Age was negatively correlated with overall and segmental methylation levels. Figure E: Single-factor correlation analysis heat map of factors affecting methylation levels in MMD group. Gender was correlated with the overall methylation level analysis of the D-loop region. F-H: Scatter plot of correlation between age and global and fragmental methylation level distribution of D-loop region in MMD group. There was no significant correlation between age and global and segmental methylation levels.

Supplementary Figure 2: D-loop methylation heatmap in MMD and non-MMD ICASO before PSM and after PSM


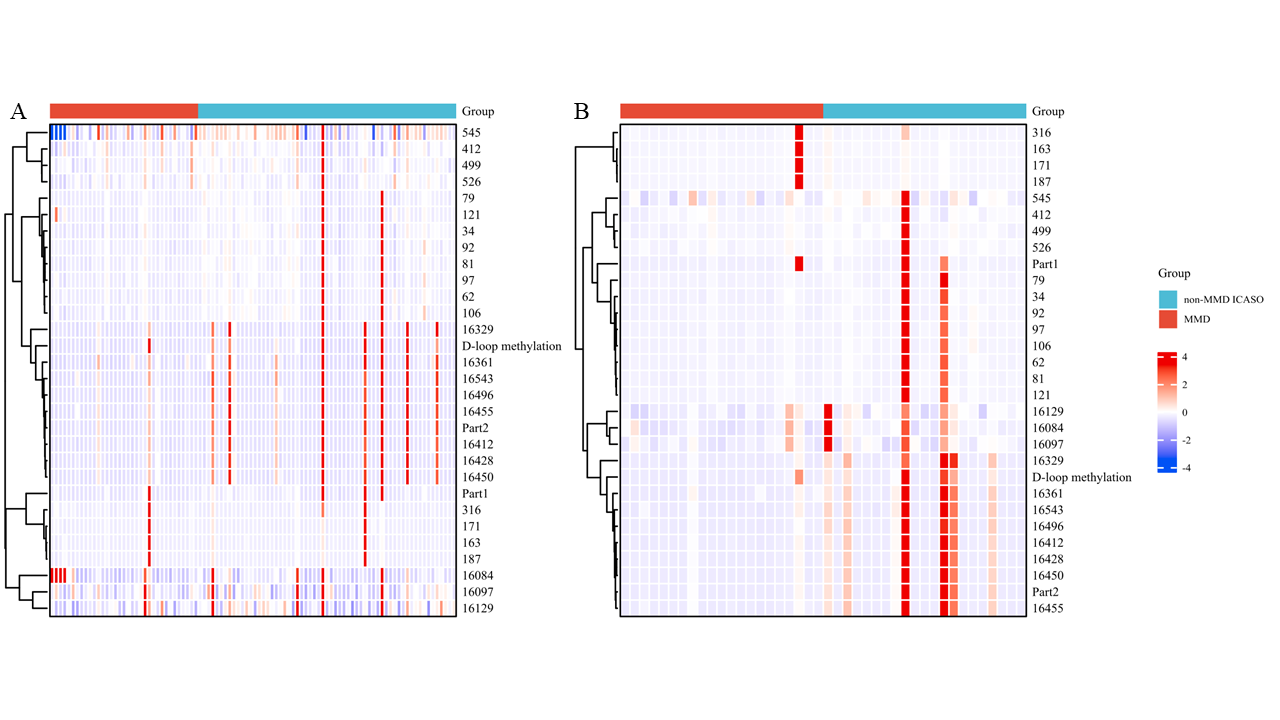


Figure A: D-loop methylation heatmap in MMD and non-MMD ICASO before PSM; Figure B: D-loop methylation heatmap in MMD and non-MMD ICASO after PSM.

Supplementary Figure 3: RNF213 knockout verification results chart


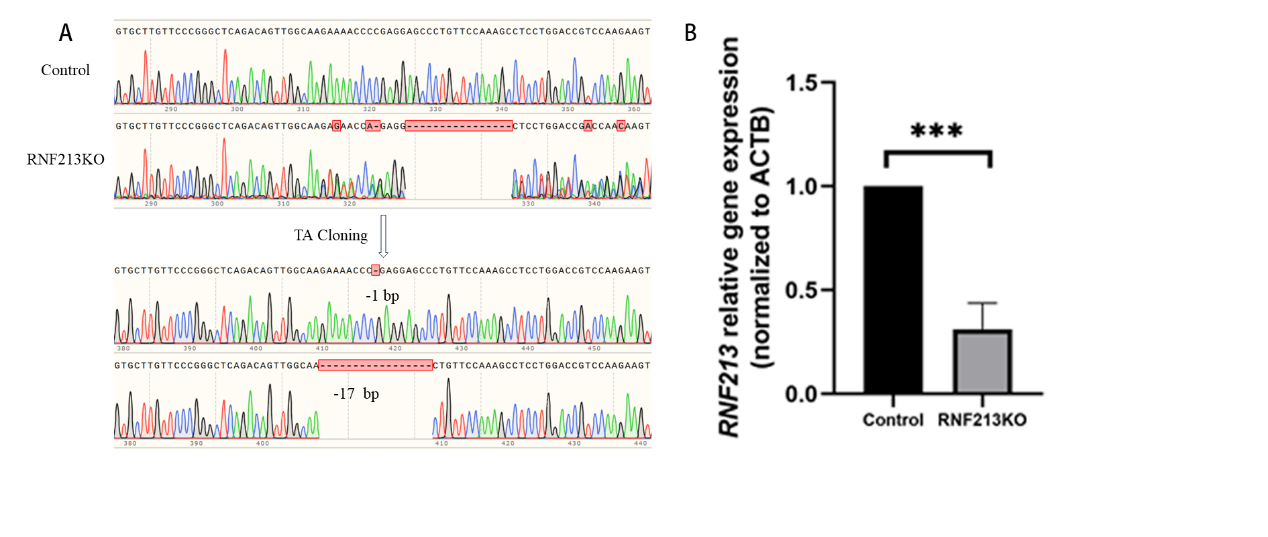


Figure A: hCMEC/D3 second-generation sequencing results: Compared with the control, the RNF3KO group showed a frameshift mutation after gene fragment knockout; TA clone sequencing found that it mainly caused mixed mutations of 1bp and 17bp deletions; Figure B: RNF213 transcription Level changes: RNF213 knockdown leads to a decrease in RNF213 mRNA transcription; ***: p＜0.001.
