# Supplementary material for: Study on Abnormal Angiogenesis in Moyamoya Disease via Mitochondrial D‐Loop Methylation
Source: Brain Behav. 2025 Nov 21;15(11):e71042. doi: 10.1002/brb3.71042 (PMC12639187; doi:10.1002/brb3.71042)
Supplement: Supplementary file 2 — Supporting Information Tables: brb371042‐sup‐0001‐tablesS1‐S5.docx [file BRB3-15-e71042-s002.docx]

Supplementary Table 1: Comparison of methylation levels between control and MMD

| Characteristics | Control | MMD | P |
| --- | --- | --- | --- |
| n | 20 | 35 |  |
| Age, median (IQR) | 44.5 (42, 47) | 45 (34.5, 51) | 0.827 |
| Male, n (%) | 10 (50%) | 14 (40%) | 0.472 |
| 34, median (IQR) | 0.010796  (0.008729, 0.013499) | 0.0076197  (0.0059674, 0.010005) | **0.001**** |
| 62, median (IQR) | 0.0062237  (0.0047928, 0.007861) | 0.0054702  (0.0039798, 0.0070071) | 0.2 |
| 79, median (IQR) | 0.0074385  (0.0059095, 0.0090686) | 0.0067389  (0.0054854, 0.0078561) | 0.233 |
| 81, median (IQR) | 0.010512  (0.0087355, 0.012202) | 0.0070093  (0.0052767, 0.0087622) | **0.002*** |
| 92, median (IQR) | 0.0094536  (0.0071961, 0.010915) | 0.0084317  (0.0070723, 0.0096151) | 0.371 |
| 97, median (IQR) | 0.007801  (0.0058025, 0.0086143) | 0.0060976  (0.0050315, 0.0075782) | 0.099 |
| 106, median (IQR) | 0.0082194  (0.0073468, 0.011128) | 0.0076401  (0.00547, 0.009819) | 0.254 |
| 121, median (IQR) | 0.0088359  (0.0065047, 0.0095126) | 0.0071669  (0.0053908, 0.0084275) | 0.114 |
| 163, median (IQR) | 0.008278  (0.0067834, 0.0099988) | 0.0061544  (0.0052226, 0.0081947) | 0.033 |
| 171, median (IQR) | 0.0073171  (0.0064837, 0.0089054) | 0.0073511  (0.0059263, 0.00869) | 0.709 |
| 187, median (IQR) | 0.0071723  (0.0057321, 0.010779) | 0.0068508  (0.0059299, 0.0085825) | 0.924 |
| 316, median (IQR) | 0.012522  (0.010303, 0.014142) | 0.013093  (0.0094217, 0.017094) | 0.828 |
| 412, median (IQR) | 0.012984  (0.011471, 0.015982) | 0.010197  (0.0081297, 0.013759) | 0.062 |
| 499, median (IQR) | 0.0081102  (0.0062413, 0.0094745) | 0.0072133  (0.006134, 0.0090491) | 0.788 |
| 526, median (IQR) | 0.0077239  (0.0062972, 0.008774) | 0.0071579  (0.0052975, 0.0091157) | 0.748 |
| 545, median (IQR) | 0.063902 (0.060428, 0.069046) | 0.053833 (0.047959, 0.065595) | **0.023*** |
| 16084, median (IQR) | 0.0064488  (0.005329, 0.0073544) | 0.0056915  (0.0045939, 0.0088655) | 0.684 |
| 16097, median (IQR) | 0.0062632  (0.0044685, 0.0092058) | 0.0066416  (0.0055722, 0.0080146) | 0.684 |
| 16129, median (IQR) | 0.0095293  (0.0074126, 0.011212) | 0.0067992  (0.0056008, 0.0090497) | **0.021*** |
| 16329, median (IQR) | 0.0063039  (0.0046506, 0.0091509) | 0.0063609  (0.0044478, 0.0085765) | 0.581 |
| 16361, median (IQR) | 0.015798  (0.013358, 0.021544) | 0.011367  (0.0091794, 0.01722) | 0.14 |
| 16412, median (IQR) | 0.0092162  (0.0074297, 0.011524) | 0.007932  (0.0061331, 0.011491) | 0.336 |
| 16428, median (IQR) | 0.0074716  (0.0057819, 0.0085252) | 0.0055866  (0.0041071, 0.0088008) | 0.164 |
| 16450, median (IQR) | 0.0066449  (0.0049413, 0.0093208) | 0.0059375  (0.00468, 0.0084995) | 0.512 |
| 16455, median (IQR) | 0.010438  (0.009273, 0.014495) | 0.0083414  (0.0056732, 0.013145) | 0.358 |
| 16496, median (IQR) | 0.0069278  (0.0061575, 0.0086678) | 0.0060519  (0.0048162, 0.0082061) | 0.119 |
| 16543, median (IQR) | 0.0081983  (0.0061753, 0.00974) | 0.0063291 (0.0050928, 0.0095949) | 0.155 |
| D-loop methylation, median (IQR) | 0.010824  (0.0087364, 0.013828) | 0.0091955  (0.008379, 0.011975) | 0.22 |
| Part1, median (IQR) | 0.0084388  (0.0074405, 0.009613) | 0.0065984  (0.0058838, 0.0087612) | **0.019*** |
| Part2, median (IQR) | 0.0086494  (0.0067081, 0.011088) | 0.0072486  (0.0063717, 0.0092031) | 0.293 |

At position34, 81, 545, 16129, and part 1 the methylation level of the healthy control was higher than that of the MMD(P<0.05). MMD: moyamoya disease; IQR: Interquartile range. *p＜0.05, **p＜0.01.

Supplementary Table 2: Comparison of methylation levels in patients with different symptoms

| Characteristics | Cerabral ishemic | Cerebral hemorrhage | Other symptom | P |
| --- | --- | --- | --- | --- |
| n | 14 | 11 | 6 |  |
| Age, median (IQR) | 41 (33.25, 50.75) | 49 (44.5, 51.5) | 44 (38.5, 46.5) | 0.365 |
| Male, n (%) | 9 (64.3%) | 7 (63.6%) | 3 (50%) | 0.796 |
| 34, median (IQR) | 0.0076744  (0.005994, 0.010191) | 0.0068027  (0.0053344, 0.0093945) | 0.0088536  (0.0086752, 0.0091888) | 0.516 |
| 62, median (IQR) | 0.0049035  (0.0039146, 0.0058416) | 0.0058714  (0.0042727, 0.0072841) | 0.0042919  (0.0040335, 0.0069642) | 0.659 |
| 79, median (IQR) | 0.0065461  (0.0052904, 0.0074707) | 0.0066566  (0.0058474, 0.0071991) | 0.008162  (0.0078372, 0.0088101) | 0.196 |
| 81, median (IQR) | 0.0071289  (0.0063546, 0.0088815) | 0.0070093  (0.0055124, 0.0082496) | 0.0099611  (0.0068326, 0.012348) | 0.516 |
| 92, median (IQR) | 0.0073832  (0.0060959, 0.0091063) | 0.0089095  (0.0079477, 0.0095158) | 0.0089916  (0.0082283, 0.010263) | 0.239 |
| 97, median (IQR) | 0.0067665  (0.0054909, 0.0075976) | 0.0058787  (0.0049454, 0.006144) | 0.0065898  (0.0055478, 0.010764) | 0.349 |
| 106, median (IQR) | 0.0080874  (0.0059901, 0.0098926) | 0.0076401  (0.0059756, 0.0080438) | 0.01011  (0.0058318, 0.014033) | 0.730 |
| 121, median (IQR) | 0.0072808  (0.0055486, 0.008109) | 0.0064935  (0.0047206, 0.0082143) | 0.0077437  (0.0064739, 0.0090313) | 0.469 |
| 163, median (IQR) | 0.006164  (0.0055772, 0.0086224) | 0.0058875  (0.0047956, 0.0080084) | 0.0063296  (0.0059677, 0.0077858) | 0.663 |
| 171, median (IQR) | 0.0059263  (0.0051436, 0.0075429) | 0.0077603  (0.0070183, 0.0083593) | 0.0080271  (0.0073733, 0.0087494) | 0.076 |
| 187, median (IQR) | 0.0066883  (0.0056785, 0.0077983) | 0.0081605  (0.0064453, 0.0088508) | 0.0073462  (0.0066632, 0.0097952) | 0.360 |
| 316, median (IQR) | 0.011022  (0.0068724, 0.01542) | 0.014583  (0.011722, 0.017973) | 0.012671  (0.010367, 0.014732) | 0.301 |
| 412, median (IQR) | 0.010472  (0.0089273, 0.013061) | 0.0109  (0.0076868, 0.013267) | 0.010338  (0.0099627, 0.017275) | 0.799 |
| 499, median (IQR) | 0.0071904  (0.0062972, 0.0088095) | 0.007091  (0.0062069, 0.0097935) | 0.007601  (0.0060987, 0.014981) | 0.954 |
| 526, median (IQR) | 0.0072859  (0.0064796, 0.0084388) | 0.0079077  (0.0066457, 0.010773) | 0.0078516  (0.0066607, 0.0094675) | 0.795 |
| 545, median (IQR) | 0.053258  (0.050382, 0.054197) | 0.059849  (0.051538, 0.069391) | 0.071804  (0.060484, 0.084664) | 0.084 |
| 16084, median (IQR) | 0.0054921  (0.0040424, 0.0071542) | 0.0055258  (0.0045939, 0.0070506) | 0.0061666  (0.0050214, 0.0069507) | 0.707 |
| 16097, median (IQR) | 0.0063182  (0.0044704, 0.0084795) | 0.0066018  (0.0057334, 0.0070606) | 0.0070941  (0.0061638, 0.0079751) | 0.552 |
| 16129, median (IQR) | 0.0085904  (0.0067762, 0.0091909) | 0.0052174  (0.0032298, 0.0067673) | 0.0091643  (0.0072832, 0.015196) | **0.012*** |
| 16329, median (IQR) | 0.0065526  (0.0037398, 0.0090546) | 0.0053248  (0.0043431, 0.0063961) | 0.0063476  (0.0056827, 0.008008) | 0.643 |
| 16361, median (IQR) | 0.014118  (0.0089599, 0.024414) | 0.011404  (0.010093, 0.014085) | 0.011698  (0.011077, 0.014209) | 0.938 |
| 16412, median (IQR) | 0.0090197  (0.0064845, 0.014641) | 0.008  (0.0072434, 0.011032) | 0.0085275  (0.0069803, 0.0096561) | 0.971 |
| 16428, median (IQR) | 0.0075456  (0.0055235, 0.01194) | 0.0052172  (0.0035438, 0.0091836) | 0.0052315  (0.0045287, 0.0055411) | 0.391 |
| 16450, median (IQR) | 0.0071323  (0.0049841, 0.010607) | 0.0053135  (0.0041771, 0.0070209) | 0.0064432  (0.0042263, 0.0083577) | 0.540 |
| 16455, median (IQR) | 0.011731  (0.0067658, 0.015548) | 0.0074786  (0.0050172, 0.013823) | 0.0097426  (0.007537, 0.011669) | 0.562 |
| 16496, median (IQR) | 0.0062423  (0.0053535, 0.011018) | 0.0050676  (0.0044639, 0.0069623) | 0.006232  (0.0044456, 0.0077437) | 0.435 |
| 16543, median (IQR) | 0.0068346  (0.0053866, 0.011318) | 0.0063935  (0.0050442, 0.0086737) | 0.0080993  (0.0057165, 0.0089099) | 0.876 |
| D-loop methylation, median (IQR) | 0.008923  (0.0082693, 0.013249) | 0.0093368  (0.0088037, 0.010774) | 0.010134  (0.008517, 0.013018) | 0.849 |
| Part1, median (IQR) | 0.0061358  (0.0058588, 0.0079648) | 0.0066394  (0.0059951, 0.0072058) | 0.0078633  (0.0065153, 0.0091577) | 0.472 |
| Part2, median (IQR) | 0.0083244  (0.0063626, 0.012686) | 0.0073454  (0.0066714, 0.0086237) | 0.0075886  (0.0065013, 0.0086793) | 0.845 |

There was no abnormality in methylation levels among MMD groups with different initial symptoms in most sites. MMD: moyamoya disease; IQR: Interquartile range. *p＜0.05, **p＜0.01.

Supplementary Table 3: Comparison of methylation levels between different gender

| Group | Characteristic | Male | Female | p |
| --- | --- | --- | --- | --- |
| ICASO | n | 63 | 33 |  |
|  | Age, median (IQR) | 52 (42.5, 56) | 52 (46, 58) | 0.61 |
|  | D-loop methylation, median (IQR) | 0.011515 (0.0092467, 0.014267) | 0.0088135 (0.0079721, 0.011447) | **0.003**** |
|  | Part1, median (IQR) | 0.0084397 (0.0066436, 0.011617) | 0.0066661 (0.0060019, 0.0087918) | **0.019*** |
|  | Part2, median (IQR) | 0.0085922 (0.0070188, 0.013916) | 0.0072881 (0.0064136, 0.008714) | **0.019*** |
| non-MMD ICASO | n | 42 | 19 |  |
|  | Age, median (IQR) | 53.5 (48.75, 58) | 57 (51.5, 69.5) | 0.117 |
|  | D-loop methylation, median (IQR) | 0.011705 (0.010307, 0.01419) | 0.0092628 (0.0079682, 0.011555) | **0.026*** |
|  | Part1, median (IQR) | 0.0091748 (0.0073474, 0.01249) | 0.0069692 (0.0062404, 0.0095715) | **0.028*** |
|  | Part2, median (IQR) | 0.0088504 (0.007425, 0.014281) | 0.0078739 (0.0069227, 0.0092154) | 0.17 |
| MMD | n | 21 | 14 |  |
|  | Age, median (IQR) | 45 (35, 51) | 47 (34.25, 50.75) | 0.987 |
|  | D-loop methylation, median (IQR) | 0.0093368 (0.0087647, 0.014208) | 0.0085223 (0.0080131, 0.010868) | **0.026*** |
|  | Part1, median (IQR) | 0.0066394 (0.0058909, 0.0091282) | 0.006543 (0.0058862, 0.0071501) | 0.654 |
|  | Part2, median (IQR) | 0.0079787 (0.0065304, 0.011003) | 0.0066461 (0.0062893, 0.0073778) | 0.089 |

When the distribution of different ages is consistent (p<0.05), male ICASO patients have higher methylation levels; the same trend is shown in the MMD and non-MMD ICASO groups. MMD: moyamoya disease; IQR: Interquartile range. *p＜0.05, **p＜0.01.

Supplementary Table 4: Comparison of methylation levels between non-MMD ICASO and MMD groups

| Characteristic | non-MMD ICASO | MMD | p |
| --- | --- | --- | --- |
| n | 21 | 21 |  |
| 34, median (IQR) | 0.011932 (0.0099786, 0.016749) | 0.0070061 (0.0057052, 0.009633) | **< 0.001***** |
| 62, median (IQR) | 0.0085128 (0.0070737, 0.012862) | 0.0054702 (0.0040984, 0.0062064) | **0.002**** |
| 79, median (IQR) | 0.0099185 (0.0073394, 0.012862) | 0.0061931 (0.0052308, 0.0081445) | **0.012*** |
| 81, median (IQR) | 0.011026 (0.0080499, 0.014323) | 0.0070093 (0.005255, 0.0090909) | **0.002**** |
| 92, median (IQR) | 0.012012 (0.01005, 0.017512) | 0.0089095 (0.0071942, 0.010488) | **0.003**** |
| 97, median (IQR) | 0.010372 (0.0091832, 0.014486) | 0.006216 (0.0058787, 0.007617) | **< 0.001***** |
| 106, median (IQR) | 0.011057 (0.0092355, 0.015312) | 0.0077459 (0.0059304, 0.0096091) | **0.004**** |
| 121, median (IQR) | 0.010459 (0.0080779, 0.012981) | 0.0073289 (0.0057484, 0.0085346) | **0.003**** |
| 163, median (IQR) | 0.0091098 (0.0075481, 0.010853) | 0.006282 (0.0053286, 0.0090847) | 0.059 |
| 171, median (IQR) | 0.0074239 (0.0060799, 0.0098915) | 0.0071453 (0.0063196, 0.0087944) | 0.921 |
| 187, median (IQR) | 0.0079015 (0.0069807, 0.010409) | 0.0069851 (0.0059915, 0.0084211) | 0.12 |
| 316, median (IQR) | 0.012664 (0.011369, 0.015211) | 0.011585 (0.0099613, 0.018201) | 0.41 |
| 412, median (IQR) | 0.012881 (0.0096521, 0.015734) | 0.010479 (0.0089076, 0.011443) | 0.16 |
| 499, median (IQR) | 0.0092251 (0.0081817, 0.012844) | 0.0071356 (0.0058168, 0.0082576) | 0.02* |
| 526, median (IQR) | 0.0091912 (0.0065717, 0.011501) | 0.0079077 (0.0067625, 0.0098765) | 0.584 |
| 545, median (IQR) | 0.068843 (0.055624, 0.073576) | 0.054934 (0.05253, 0.069064) | 0.102 |
| 16084, median (IQR) | 0.0078995 (0.007326, 0.01026) | 0.0055038 (0.0039442, 0.007329) | **0.007**** |
| 16097, median (IQR) | 0.0087413 (0.0052493, 0.010764) | 0.0066018 (0.0055489, 0.0086744) | 0.343 |
| 16129, median (IQR) | 0.010551 (0.0079051, 0.013082) | 0.007586 (0.0065453, 0.0095321) | 0.07 |
| 16329, median (IQR) | 0.007764 (0.0068102, 0.133) | 0.0053248 (0.0038067, 0.0071942) | **0.002**** |
| 16361, median (IQR) | 0.024179 (0.012964, 0.15591) | 0.012028 (0.010542, 0.020737) | **0.017*** |
| 16412, median (IQR) | 0.012436 (0.0086295, 0.15075) | 0.0097185 (0.0065749, 0.015152) | 0.125 |
| 16428, median (IQR) | 0.0074808 (0.0060274, 0.1393) | 0.0054046 (0.0037469, 0.01261) | **0.015*** |
| 16450, median (IQR) | 0.0072337 (0.0064424, 0.14) | 0.005069 (0.0040912, 0.010923) | **0.024*** |
| 16455, median (IQR) | 0.0099064 (0.0082448, 0.15347) | 0.0091935 (0.0059356, 0.015899) | 0.087 |
| 16496, median (IQR) | 0.0088094 (0.0065267, 0.16832) | 0.0058561 (0.0047297, 0.011628) | **0.036*** |
| 16543, median (IQR) | 0.0092791 (0.0070454, 0.15075) | 0.0063291 (0.0050722, 0.011628) | 0.061 |
| D-loop methylation, median (IQR) | 0.011929 (0.010586, 0.066753) | 0.0090813 (0.0084757, 0.011814) | **0.006**** |
| Part1, median (IQR) | 0.0096044 (0.0087918, 0.014118) | 0.0064404 (0.0059144, 0.0091282) | **< 0.001***** |
| Part2, median (IQR) | 0.0093949 (0.0082873, 0.14894) | 0.0076663 (0.0064734, 0.013246) | **0.012**** |

The methylation levels of 34, 62, 79, 81, 92, 97, 106, 121, 499, 545, 16084, 16329, 16361, 16428, 16450, 16496, Part1, Part2 and overall in the patient group were higher than those in the MMD group (p < 0.05). The rest of the site did not see obvious difference.MMD：moyamoya disease；ICASO：Intracranial major artery；IQR：Interquartile range. *p＜0.05, **p＜0.01,***p＜0.001.

Supplementary Table 5: Comparison of methylation levels in patients with and without RNF213 p.R4810K MMD

| Characteristic | With 4810K | Without 4810K | p |
| --- | --- | --- | --- |
| n | 3 | 3 |  |
| Age, mean ± sd | 29.6667±10.3333 | 30±10.0167 | 0.980 |
| Male，% | 1(33.3333%) | 2(66.6667%) | 0.567 |
| 34, mean ± sd | 0.0079±0.0023 | 0.0111±0.0017 | 0.319 |
| 62, mean ± sd | 0.0061±0.0013 | 0.0061±0.0017 | 0.998 |
| 79, mean ± sd | 0.0063±0.0008 | 0.0085±0.0007 | 0.099 |
| 81, mean ± sd | 0.008±0.0009 | 0.0101±0.0018 | 0.360 |
| 92, mean ± sd | 0.0065±0.002 | 0.009±0.0011 | 0.324 |
| 97, mean ± sd | 0.0068±0.0007 | 0.0085±0.0028 | 0.587 |
| 106, mean ± sd | 0.0073±0.0017 | 0.0106±0.0015 | 0.222 |
| 121, mean ± sd | 0.0056±0.0014 | 0.0093±0.0032 | 0.355 |
| 163, mean ± sd | 0.0076±0.0005 | 0.0131±0.0039 | 0.234 |
| 171, mean ± sd | 0.008±0.0004 | 0.0124±0.0045 | 0.389 |
| 187, mean ± sd | 0.0075±0.0005 | 0.0113±0.0038 | 0.385 |
| 316, mean ± sd | 0.0134±0.0017 | 0.0153±0.0073 | 0.805 |
| 412, mean ± sd | 0.013±0.0015 | 0.0162±0.0062 | 0.662 |
| 499, mean ± sd | 0.0088±0.0017 | 0.0137±0.0068 | 0.521 |
| 526, mean ± sd | 0.0067±0.0009 | 0.0138±0.0054 | 0.323 |
| 545, mean ± sd | 0.0817±0.0117 | 0.0477±0.006 | 0.061 |
| 16084, mean ± sd | 0.007±0.0002 | 0.0072±0.0009 | 0.886 |
| 16097, mean ± sd | 0.0076±0.0003 | 0.0078±0.0015 | 0.939 |
| 16129, mean ± sd | 0.0044±0.0012 | 0.0079±0.001 | 0.089 |
| 16329, mean ± sd | 0.0059±0.0003 | 0.0083±0.0027 | 0.457 |
| 16361, mean ± sd | 0.0117±0.0002 | 0.015±0.0027 | 0.357 |
| 16412, mean ± sd | 0.0088±0.0021 | 0.0122±0.0032 | 0.422 |
| 16428, mean ± sd | 0.0067±0.0008 | 0.0085±0.0029 | 0.592 |
| 16450, mean ± sd | 0.0069±0.0014 | 0.0089±0.0015 | 0.402 |
| 16455, mean ± sd | 0.008±0.0019 | 0.0108±0.0034 | 0.517 |
| 16496, mean ± sd | 0.0048±0.0008 | 0.007±0.0013 | 0.199 |
| 16543, mean ± sd | 0.0071±0.0011 | 0.0082±0.0021 | 0.664 |
| D-loop methylation, mean ± sd | 0.0104±0.001 | 0.0118±0.0022 | 0.586 |
| Part1, mean ± sd | 0.007±0.0007 | 0.001±0.0021 | 0.249 |
| Part2, mean ± sd | 0.0075±0.0008 | 0.0099±0.0024 | 0.399 |

The D-loop methylation of most sites in patients carrying the mutation were greater than those without the mutation, but the difference was not statistically significant (p＞0.05).
